# 

**Published:** 1868-09

**Authors:** 


NOTICE.—The Mad River Valley Dental Society will hold its next regular meeting in Dayton, O., Oct. 6, (the first Tuesday) at 10 A. M. B. F. ROSSON, *Pres.*, Troy.

G. L. PAINE, *Sec.*, Xenia.

### OHIO DENTAL COLLEGE SESSION.

The twenty-third annual session of this institution will be opened on the 15th of October, proximo, when, we trust, that all who intend to enter the classes will be present so far as possible. The regular course of instruction will then commence, and subjects once considered cannot be again taken up, so that, so far as any fail to enter at the beginning, they sustain a clear loss, and one that they cannot retrieve during the session.

No effort will be spared to make the course as thorough as possible, and with the present classification and arrangements, much more can be done than by the former method.

We, therefore, suggest that all who shall decide to enter will do so promptly. T.

### The Dental Register

OF THE WEST.

*Issued Monthly, at $3 a Year in Advance.*

DEVOTED TO THE INTERESTS OF THE DENTAL PROFESSION.

EDITED BY J. TAFT AND G. WATT.

The volume commences in January and closes in December.

The Register being issued monthly is always fresh, therefore indispensable to the practitioner who desires to keep posted up with the new inventions and improvements which are daily developed. Neither expense nor effort will be spared to give value and variety to its contents. Articles will be illustrated with well executed engravings whenever they will add to the interest or assist in explanation.

Its extensive circulation and frequency of issue makes it one of the BEST DENTAL ADVERTISING MEDIUMS in the United States.

RATES OF ADVERTISING.

One page, 1 year, $50. Half page, 1 year, $30. Quarter page, or card, 1 year, $20.

All advertising bills payable in advance, or at furthest after the issue of the first number containing the advertisement. All transient advertisements to be paid for in advance.

☞ CONTRIBUTIONS SOLICITED.

Address, J. TAFT, or "DENTAL REGISTER," Cincinnati Ohio.

Business Communications to

**J. TAFT, Publisher,**

No. 238 Fourth St., between Plum and Central Avenue

